# The Rise, Progress and Present Status of Dentistry

**Published:** 1867-11

**Authors:** H. F. Bishop

**Affiliations:** Worcester, Mass.


(To be Continued.)

---

### ARTICLE V.

### *The Rise, Progress and Present Status of Dentistry.*

By H. F. Bishop, D.D.S., of Worcester, Mass.

Mr. President and gentlemen of the Mass. Society of Dental Surgeons.—To-day—Time, that ceaseless traveller wafts us by another land mark ; to-day brings us one year nearer our final home, to-day, we have our third anniversary and commence our fourth year of existence. Our kind Heavenly Father has dealt gently with all our members ; none have been called the past year to walk the golden street of that bright realm where no pain exists—where neither moth nor rust doth corrupt, but still the unbroken band are toiling and battling with pain, decay and corruption, to keep their destructiveness in check and alleviate the suffering and waste of human life. Two years ago our worthy President addressed us upon the aims and duties of the profession; in which able address we were reminded of our obligations to our families and dependants as well as the great duty we owe to *our-selves* in order to maintain our usefulness and be thus enabled to do our whole duty. A year since, we listened to our first Vice-President upon the past and future of the dental profession, when many incentives were held out for its elevation, while we are transmitting it to our successors.

In reviewing the present status of Dentistry, let us hastily take a chronological survey of its rise and progress, and then consider the important developements of the past year, as well as notice the increasing claims the science is making upon an appreciative public.

How long has dentistry been practiced? Who first substituted artificial teeth for the loss of natural ones? Who discovered the utility of filling teeth for their preservation? These are questions often asked and not so easily answered. Dentistry, it has been suggested originated almost at the cradle of the human race, yet comparatively little can be said of its importance in the scientific world till the present century. Mankind from the first-born—Cain and Abel, have been endowed with the rudiments of twenty deciduous and thirty-two permanent teeth —all of which are subject to disease and accident; hence the dental doctor. From the days of Adam (the only man who never had infant teeth) living in the first ten centuries to the days of Methusaleh who died the year Noah entered the ark, and existed in the last ten centuries before the flood, little or nothing can now throw light on their time; we can only presume that our art could scarcely have been been needed in those early days of the world's freshness.

Hippocrates born four hundred years before Christ, alludes to the inserting of teeth fastened by gold wire; Celsus a physician in the first century, describes the extraction of teeth and other operations; Galen the celebrated Greek physician flourished in the second century; he was appointed medical superintendent of the gladiators in his native city, in which melancholy duty it is to be supposed he must have gained some knowledge of the care of wounds, and possibly some experience might have given him new dentalogical facts. The Emperor Marcus Aurelius placed his son Commodus, a tender youth of nine years old, under his care—but he has failed to tell us the condition of his six year old molars, whether they were saved or were extracted. Whether the Egyptians and Brahmins, once the polished nations of the earth, were skilled or not in dentistry, must remain a matter of conjecture, since all traces of their learning and ancient history were lost when the grand library at Alexandria was burned about the year 640. If stuffed teeth have been found in mummies, we have no certainty of the operation being performed on the living subject, but it may have been introduced by the embalmer to prevent further decomposition.

It is supposed that Albucasis was the first to suggest the supplying of lost teeth by other human teeth or those of animals, or by artificial ones of bone or ivory.

Following on up the historical hill of time from the destruction of the library in the 9th century and the dark ages following, we come to the 16th century—the art of printing having in the meantime come to the aid of science. Eutachius the distinguished Italian anatomist flourished at this time and published the first work of any consequence on dentistry; and Ambrose Paré describes the manner of replacing the teeth and the use of obturators.

*Taking a survey of the world in the 16th century at it close, say one hundred years after Columbus discovered our continent, Southern Europe had decidedly the advance of all other countries in knowledge and acquaintance with the sciences—Spain and Italy taking the lead. In the 17th century publications gradually multiply, and looking northward on the continent of Europe we see new lights—France has its Muller, Martin, and a few others. Germany is well represented by several works published at Leipsic—Switzerland has its celebratedRyff—Austria and Prussia publish works of which some appertain indirectly to dental surgery. Up to this period we recognize gratefully the influence of medical and dental literature upon the profession, but not until the 18th century do we find many practical dentists who have born our science along as a speciality. Then, France getting the lead had scores of able men in the field, many of whose works are useful at the present day† :—the distinguished dentists were quite generally writers upon the science of their profession, and it is certainly singular that with so much talent and the growing light on the subject, a higher standard should not have earlier been established. The first idea of porcelain teeth is due to a French apothecary as early as 1774—having an ivory set which gave him great inconvenience, he experimented until with the help of a chemist and porcelain manufacturers of Paris, he succeeded in making a porcelain of a grayish color, which shrunk very little. In 1776 he communicated his secret to the French Academy and was elected a member in consequence. But it was Musseer de Chemant a distinguished dentist of Paris who improved these teeth and

---

*Both Arculanus and DeVigo about this time filled teeth with reference to temperature—the latter using gold leaf it is said. Thus stuffing the teeth for alleviating pain, gradually led the way to plugging them with metal for preservation.

†Jourdain, Beaupeau, Fauchard, Bunon, and Bordet, all lived and flourished at this period.

obtained a patent from Louis XVIth for the exclusive right to make and set them ; he encountered however violent opposition from French dentists in their introduction.*

About this time England begins to have equal claims upon our attention: she had in the field Hunter who, though not a practical dentist, is entitled to our great admiration and respect. Blake, Curtis, Berdmore, who was dentist to George the Third, Woofendale and others. The latter was one of the few Europeans who came to America and established a fame upon both continents—a compliment which Americans have paid with interest in the 10th century. Just one hundred years ago (from 1766 to 1768) Robert Woofendale a pupil of Berdmore practised in the city of New York for two years—the first *regular* dentist it is said in this country. Near the close of the century he returned to America practising again a few years in New York, and then leaving his business to his son John.

John Woofendale was the first dentist of whom my childish ears heard, his services having been called in the early part of the present century to set teeth for my father ; the said teeth were calves' teeth reduced in size by a file and set on pivots of wood ; and they were afterwards supplanted when necessity required, by the ingenuity of the patient himself, who subsequently related the incident to his children, four of whom became dentists.

As early as 1784 Dr. James Gardette, a Frenchman by birth was established in Philadelphia after a succession of trials for business at Newport, Boston and New York, and remained in practice there forty-five years. He was the first to apply the principle of suction or atmospheric air to sustain artificial teeth in the mouth, which he discovered as early as 1800. He was one of the earliest to adopt gold for filling instead of lead and tin then in use,

---

*And it was many years before they came into general use—Europeans still adhering to ivory and hippotamus tusk, both of which have been used by them to some extent, up to a very recent date.

preparing his gold himself from Dutch ducats. His valuable discoveries attracted the attention of distinguished men in the profession in France, and other countries.

Isaac Greenwood the son of a professor in Harvard College practiced in Boston for a long time, and his son, John Greenwood was said to be the only dentist in New York in 1790. He struck up a gold plate by swaging for the base of artificial teeth in 1799, and claimed to be the first person who had done so in America. He was Washington's dentist and made him several sets of teeth.*

(To be Continued.)

*The estimation in which he was held by Washington, may be seen by the Washington letter in Harris' Dental Dictionary.

