# Errata

**Published:** 1867-12

**Authors:** 


*Errata*—In the first part of Dr. H. F. Bishop's Address on *Rise, Progress, &c. of Dentistry*, in November No. of Journal the following typographical error occurs on Page 350, "Musseer" for "Monsieur."

